# Correlation between blood telomere length and CD4+ CD8+ T-cell subsets changes 96 weeks after initiation of antiretroviral therapy in HIV-1–positive individuals

**DOI:** 10.1371/journal.pone.0230772

**Published:** 2020-04-08

**Authors:** Mathieu Chalouni, Javier Rodriguez-Centeno, Assia Samri, Julian Blanco, Natalia Stella-Ascariz, Cedrick Wallet, Hernando Knobel, David Zucman, Belen Alejos Ferreras, Brigitte Autran, Rodolphe Thiebaut, François Raffi, Jose Ramon Arribas, on behalf of the NEAT 001/ANRS 143 Trial Study Group

**Affiliations:** 1 ISPED, Inserm Bordeaux Population Health, Univ Bordeaux, UMR 1219, Bordeaux, France; 2 Hospital La Paz Institute for Health Research, Madrid, Spain; 3 Center for Immunology and Microbial Infections—CIMI-Paris, Sorbonne Universités, INSERM U1135, Paris, France; 4 IrsiCaixa AIDS Research Institute, Institut de Recerca Germans Trias i Pujol, Germans Trias i Pujol University Hospital, Badalona, Barcelona, Spain; 5 Inserm U897 Epidemiologie-Biostatistique, University of Bordeaux, Bordeaux, France; 6 Hospital del Mar, Barcelona, Spain; 7 Hôpital Foch, Service de Médecine Interne, Suresnes, France; 8 National Center of Epidemiology, Carlos III Health Institute, Madrid, Spain; 9 Centre de Recherches en Immunologie et Maladies Infectieus, Inserm UMR-S 1135, CIMI-Paris, Université Pierre et Marie Curie, Paris, France; 10 Infectious Diseases Department, University of Nantes, Nantes, France; Uniformed Services University, UNITED STATES

## Abstract

In 31 participants who started first-line antiretroviral therapy in the NEAT 001/ANRS 143 clinical trial, we found after 96 weeks a statistically significant increase in blood telomere length (TL) of 0.04 (T/S Ratio) (p = 0.03). This increase was positively correlated with both the change in the percentage of CD4^+^ T-cells and with the decrease of CD38^+^ molecules on Central Memory CD8^+^ and negatively correlated with the change in the percentage of CD4^+^ Effector Memory cells. Increase in TL could be an expression of immune reconstitution and the associated decrease in immune activation. We acknowledge for the low statistical power due to the small sample size and the potential for false positive results due to multiple testing. Hence, further studies are needed to confirm these observations.

## Introduction

Untreated HIV infection causes an accelerated aging of the human immune system, an alteration also known as immunosenescence. HIV associated immunosenescence shares many characteristics inherent to the normal aging of the human immune system [1]: reduced thymic function, low naïve/memory cell ratio, low CD4^+^/CD8^+^ ratio, a shift of the maturation of T-cells towards phenotypes of limited proliferative potential (CD27^-^ CD28^-^) with short telomeres and increased expression of the immunosenescence marker CD57. Consequently, untreated HIV infected persons have shorter blood telomere length (TL) than age-matched uninfected controls [2].

In the NEAT 001/ANRS 143 study, a randomised clinical trial that showed non-inferiority over 96 weeks of boosted darunavir/ritonavir plus raltegravir vs boosted darunavir/ritonavir (DRV/r) plus tenofovir disoproxil fumarate (TDF)/emtricitabine (FTC) in 805 antiretroviral naïve HIV-infected adults [3], we have reported a significant increase of blood TL after 96 weeks of follow-up, with a significant higher gain in blood TL in participants receiving boosted darunavir/ritonavir plus TDF/FTC compared to those receiving boosted darunavir/ritonavir plus raltegravir [4]. Our hypothesis to explain this increase in blood TL is that blood TL represents a marker of an immune reconstitution phenomenon in which T cell populations shift back towards less differentiated phenotypes with higher replicative potential and longer telomeres. In order to test our hypothesis, we have analyzed the association of TL changes after 96 weeks of initial ART with changes in T cell subpopulations in a subgroup of participants of the NEAT 001/ANRS 143 trial.

## Materials and methods

NEAT 001/ANRS 143 [3] was a randomised 1:1, open-label, 96-week, non-inferiority trial conducted in 78 clinical sites in 15 European countries between August 2010 and October 2013. The study was approved by the Clinical Research Ethics Committee of the La Paz University Hospital in accordance with the principles of the Declaration of Helsinki. All trial participants were over 18 and gave written informed consent. Inclusion criteria were: HIV RNA greater than 1000 copies per mL and CD4 cell count under 500 cells per μL in ART-naive participants and no evidence of major International Antiretroviral Society-USA resistance mutations (the full study design and patient population have been previously described) [3]. Exclusion criteria were: receiving treatment for mycobacteriosis or malignant disease, tested positive for hepatitis B virus surface antigen, pregnancy and estimated creatinine clearance of less than 60 mL per min or any other relevant laboratory abnormalities.

For the present analysis we have selected participants from the Viral and Immunologic Dynamics and Inflammation substudy (NEAT-VIDI) with measurement of TL and at least one T cell marker at ART treatment initiation and 96 weeks later. The NEAT-VIDI substudy included 63 participants. For the present analysis 26 participants were excluded because appropriate blood samples were not available and 6 because flow cytometry was not performed. Participants excluded were compared to included participants using Student tests for quantitative variables and Fisher’s exact test for categorical variables.

The outcome was the correlation between TL, expressed as ratio of telomere (T) to single-copy gene (S), measured with qPCR as previously described [5] and T cells markers. T cells markers studied were the percentages of CD4^+^ and CD8^+^ T cells, percentages of CD45RA^+^CCR7^+^ naïve (N), CD45RA^-^CCR7^+^ central memory (CM), CD45RA^-^CCR7^-^ effector (E), CD45RA^-^CCR7^-^CD27^-^ effector memory (EM), CD45RA^-^CCR7^-^CD27^+^ transitional memory (TM) and CD45RA^+^CCR7^-^ terminal effector memory (TEMRA) cells in CD4^+^ and CD8^+^ T cells, the percentages of activation markers: HLADR^+^, CCR5^+^ and HLADR^+^ CCR5^+^ on the above defined CD4^+^ T cell subsets, and the percentages of HLADR^+^, CD38^+^, CCR5^+^, HLADR^+^ CD38^+^, HLADR^+^ CCR5^+^, CD38^+^ CCR5^+^, CD38^+^ CCR5^+^ HLADR^+^, the value of MFI of CD38^+^ and the number of CD38^+^ PE molecules on CD8^+^ T cell subsets. The following antibodies were used: CD3-APCCy7, CD4-Percp, CD8-BV421, CD45RA-FITC, CCR7-PeCy7, CD27-BV605. The expression of CCR5 co-receptor was done using CCR5-PE-CF594 or CCR5-PE. Immune activation was analyzed in parallel by using the anti-HLA-DR-APC, CD38-PE. We used BD Quantibrite^™^ PE beads kit (BD Biosciences, San Jose, CA, USA) to directly quantify the number of CD38 molecules on CD8 T cells by converting fluorescence data [6] according to the recommendations of the manufacturer. Briefly, 2 million thawed cells were incubated with the Fixable viability Dye efluor 506 (eBioscience, San Diego, California, USA) on ice for 30 minutes prior to membrane staining, then washed and incubated on ice for 20 minutes with monoclonal antibodies, then washed and fixed (BD Cellfix) (Becton-Dickinson, Franklin Lakes, New Jersey, USA). The cells were acquired on a 5-laser-beam LSR-Fortessa device or a FACS-Canto II) (Becton-Dickinson, Franklin Lakes, New Jersey, USA) and the standardized analysis was done using the FacsDIVA version 6.1.3 software. The median percentage of living lymphocytes acquired was 92. The associations between TL changes and T cell marker values at 96 weeks after the ARV treatment initiation were estimated by a linear model adjusted for TL value at week 0. For markers which were significantly associated with TL evolution, associations between TL changes and T cell marker values 96 weeks after the ARV treatment initiation were estimated separately in each treatment group by linear model adjusted for TL value at week 0 and an interaction between T cell marker evolution and treatment arm. To take into account multiplicity of statistical tests, the false discovery rate (FDR) and the adjusted p-values were calculated in addition to raw p-values [7].

## Results

Thirty-one participants from the NEAT-VIDI trial had TL measurement at treatment initiation and at week 96 and at least one T cell marker value at treatment initiation and at week 96. Baseline characteristics of the participants are described in Table 1. Mode of HIV transmission was significantly different between included participants and participants excluded due to inappropriate blood samples or flow cytometry was not performed were frequently. No other differences were found between included and excluded participants. In both groups, no participants were co-infected by hepatitis C virus. At week 96, 28 (90.3%) of the participants had achieved a plasma HIV-RNA viral load of less than 50 copies/mL. Mean change in CD4^+^ T cell count was 158 per mm^3^ and mean change in CD4^+^/CD8^+^ ratio was +0.38.

**Table 1:** Characteristics at baseline of the participants from the NEAT-VIDI substudy (n = 31) and NEAT 001/ANRS 143 trial (n = 805).

	VIDI substudy patients (n = 63)			
Characteristics *	VIDI-TL substudy (n = 31)	Excluded patients (n = 32)	p-value	Full NEAT 001/ANRS143 trial (n = 805)
**Gender**				
Female	3 (10%)	3 (10%)	1.00	95 (12%)
**Age (years)**	43 [34; 48]	40 [33; 45]	0.32	38 [31; 46]
**Ethnicity**			0.68	
Missing	0	6		
Asian	1 (3%)	1 (4%)		10 (2%)
Black	6 (19%)	2 (8%)		101 (13%)
Caucasian	22 (71%)	22 (84.6%)		658 (82%)
Other	2 (6%)	1 (4%)		27 (3%)
**Mode HIV transmission**			0.04	
Missing	4	2		38
Homosexual/bisexual	15 (56%)	25 (83%)		544 (71%)
IVDU	0 (0%)	0 (0%)		13 (2%)
Heterosexual	10 (37%)	5 (17%)		195 (25%)
Blood transfusion/haemophiliac	0 (0%)	0 (0%)		1 (<1%)
Other	2 (7%)	0 (0%)		7 (1%)
**Time since HIV diagnosis (years)**	1.0 [0.2; 3.1]	1.2 [0.3 ; 2.3]	0.41	1.1 [0.3; 3.2]
**HIV CDC clinical stage**			0.83	
A	27 (87%)	29 (91%)		666 (83%)
B	3 (10%)	3 (9%)		101 (12%)
C	1 (3%)	1 (0%)		38 (5%)
**CD4 cell count (cells per μL)**	311 [216; 436]	378 [263; 434]	0.16	333 [253; 399]
**CD8 cell count (cells per μL)**	810 [604; 1310]	934 [661; 1299]	0.93	848 [617; 1152]
**CD4/CD8 ratio**	0.33 [0.21; 0.61]	0.39 [0.24; 0.62]	0.31	0.37 [0.24; 0.51]
**HIV-1 RNA (log10 cop/mL)**	4.8 [3.8; 5.2]	5.1 [4.5; 5.3]	0.07	4.8 [4.3; 5.2]
**ART regimen**			1.00	
DRV/r–RAL	15 (48%)	15 (47%)		401 (50%)
DRV/r–TDV/FTC	16 (52%)	17 (53%)		404 (50%)

* Median [IQR] or n (%)

**Fig 1:**
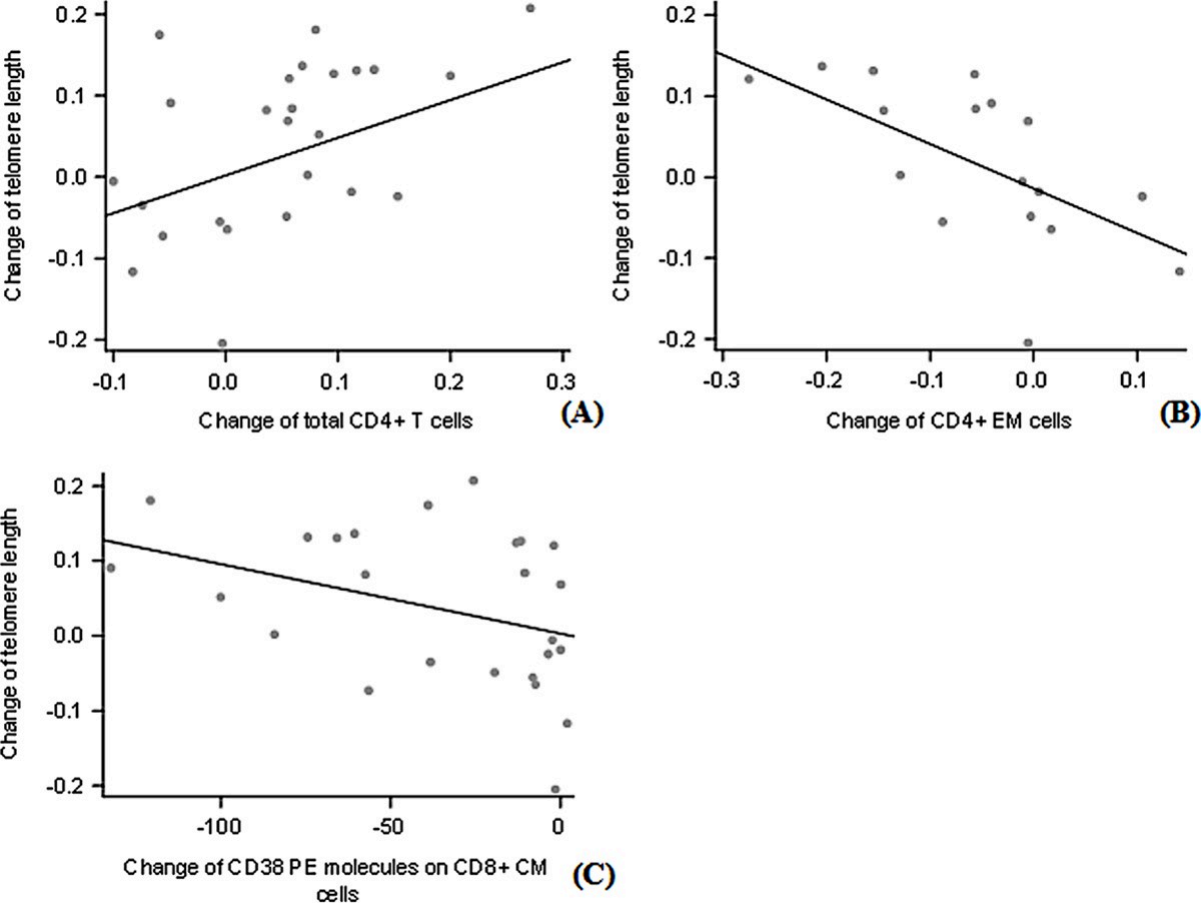
Correlation between baseline and 96 weeks after antiretroviral treatment initiation change in telomere length and change of the percentage of T cells subsets in participants from the VIDI substudy of NEAT 001 / ANRS 143 trial. Percentage of the CD4^+^ T cells (A), percentage of CD4^+^ effector memory (EM) cells (B) and the number of the CD38^+^ PE molecules on CD8^+^ central memory (CM) cells (C).

The mean blood TL between week 0 and week 96 had a statistically significant increase of 0.04 (T/S Ratio) (SD 0.10. raw p-value = 0.03). Adjusted for telomere length at week 0, treatment with DRV/r + RAL was associated with an increase of telomere length at week 96 of 0.05 higher than participants treated with DRV/r + TDV/FTC, this difference was not significant (p = 0.1186). In the CD4^+^ T cell population, the change in TL between treatment initiation and week 96 was significantly correlated with the change in the percentage of total CD4^+^ T cell in the same time period. For an increase of 100 percent of CD4^+^ T cell, TL increases by 0.46 (T/S Ratio) (p = 0.03, FDR-adjusted p-value: 0.76) (Fig 1A). On the contrary, the change in the TL was negatively correlated with the change in the percentage of CD4^+^ EM cells. Indeed, for an increase of 100 percent of CD4^+^ EM cells, the TL decreases by 0.55 (T/S Ratio) (p = 0.01, FDR-adjusted p-value: 0.76) (Fig 1B). Trends for a positive correlation between the change in the TL and the change in the percentage of CD4^+^ TEMRA cells (p = 0.05, FDR-adjusted p-value: 0.76) and for a negative correlation with the change in the percentage of CD4^+^ E cells (p = 0.06, FDR-adjusted p-value: 0.76) were also found. No statistically significant association was found among other T cells markers from the CD4^+^ population and the telomere length evolution.

For the CD8^+^ population, a significant negative correlation with the TL change was found only with the change in the number of CD38^+^ molecules on CD8^+^ CM cells. For a decrease of 10,000 CD38^+^ molecules on CD8^+^ CM cells the TL increased by 0.09 (T/S Ratio) between treatment initiation and week 96 (p = 0.05, FDR-adjusted p-value: 0.76) (Fig 1C). We also found trends toward negative correlations between the change in the TL and the changes in the percentages of total CD8^+^ T cells (p = 0.10, FDR-adjusted p-value: 0.77), of CD38^+^ CD8^+^ T cells (p = 0.08, FDR-adjusted p-value: 0.77), of HLADR^+^-CD38^+^ CD8^+^ Naïve T cells (p = 0.06, FDR-adjusted p-value: 0.76), of CD38^+^ CCR5^+^ HLADR^+^ CD8^+^ CM cells (p = 0.07, FDR-adjusted p-value: 0.76), the number of CD38^+^ molecules on CD8^+^ E cells (p = 0.06, FDR-adjusted p-value: 0.76) and on CD8^+^ TM cells (p = 0.08, FDR-adjusted p-value: 0.77). After controlling for FDR, no statistically significant association between TL changes and T-cells markers changes were found.

Table 2 shows correlations among T cells markers and TL stratified by treatment group. For this analysis we have selected markers that in the overall population were significantly associated with TL change or were close to significance. For the CD8^+^ population, a significant negative correlation (p = 0.04) with the TL change was found only with the change in total CD8^+^ T cells in participants receiving DRV/r-RAL.

**Table 2:** Correlation between change of the CD4 and CD8 population markers and change in telomere length between W00 and W96 according to treatment group, n = 31.

	DRV/r-RAL (n = 15)	DRV/r-TDF/FTC (n = 16)	
T cell population	TL change for an increase of 100 units of the marker* [95% confidence interval]		p-value interaction
**CD4**^**+**^ **(%)**			
Total CD4^+^	+0.47 [-0.12 ; 1.05]	+0.46 [-0.25 ; 1.16]	0.72
E cells	-0.49 [-1.01 ; 0.04]	-0.18 [-0.67 ; 0.30]	0.35
EM cells	-0.53 [-1.16 ; 0.10]	-0.95 [-2.15 ; 0.25]	0.85
TEMRA cells	+0.35 [-0.56 ; 1.26]	+0.18 [-0.12 ; 0.47]	0.70
**CD8**^**+**^ **(%)**			
Total CD8^+^	-0.60 [-1.17 ; 0.02]	-0.23 [-1.15 ; 0.69]	0.35
CD38^+^	-0.20 [-0.54 ; 0.14]	-0.23 [-0.63 ; 0.17]	0.39
HLA-DR^+^ CD38^+^ Naïve cells	-1.29 [-2.88 ; 0.31]	-2.07 [-7.36 ; 3.21]	0.77
CD38^+^ PE molecules on CM cells	-0.001 [-0.002 ; 0.001]	-0.002 [-0.005 ; 0.001]	0.38
CD38^+^ CCR5+ HLA-DR^+^ CM cells	-0.38 [-0.81 ; 0.04]	-0.69 [-3.13 ; 1.76]	0.69
CD38^+^ PE molecules on E cells	-0.001 [-0.004; 0.02]	-0.003 [-0.007; 0.001]	0.83
CD38^+^ PE molecules on TM cells	-0.001 [-0.005; 0.002]	-0.008 [-0.018; 0.001]	0.77

* Adjusted by telomere length at week 0

Sensitivity analyzes were realised to take into account age as a confounding factor in the association between T cell markers change and telomere length change between W0 and W96 (Supplementary material) and results were very closed to those without adjustment on age at baseline.

## Discussion

In this VIDI substudy of the NEAT 001/ANRS 143 clinical trial we have found that changes in blood TL occurring 96 weeks after initiation of antiretroviral therapy (ART) were correlated positively with the change in the percentage of CD4^+^ on lymphocytes and negatively with the change in the percentage of CD4^+^ EM cells. This is the first time that the correlation between blood TL and T cell subpopulations has been evaluated in a clinical trial of ART naïve participants using contemporary ART. Compared to baseline, mean blood TL increased significantly at week 96 in the participants included in the VIDI substudy. In two other different datasets we have found progressive increases of blood TL in HIV infected adults receiving effective ART [4,8]. First, in the NEAT 001/ ANRS 143 TL substudy we have found a statistically significant increase in mean blood TL up to 96 weeks after ART initiation in the 201 participants included [4]. Second, in a cohort of virologically suppressed HIV infected participants we have also found that mean blood TL increased significantly after two years of follow-up [8]. Two older studies [9,10] had also found an increase in T cell TL 24 and 48 weeks after ART. These increases in blood TL after initiation of ART are contrary to studies performed in the general population, in which mean blood TL measured by qPCR analysis shows annual decrease [11].

Our results support the hypothesis that the increase in blood TL occurring in HIV infected persons receiving efficacious ART is a marker of immune reconstitution [4]. We found that increases in blood TL correlated positively with the change in the percentage of total CD4^+^ T cells and negatively with the percentage of CD4^+^ EM cells. After ART initiation there is a decrease of antigenic stimulation secondary to rapid control of HIV replication and a shift towards T-cell subpopulations with longer TL (naïve and central memory). The positive correlation between blood TL and change in the percentage of total CD4^+^ T cell might be the reflection of new circulation of undifferentiated CD4^+^ cells with long telomeres. The negative correlation between blood TL and the percentage of CD4+ EM cells is also in agreement with a decrease in antigenic stimulation that drives lymphocyte differentiation towards phenotypes with short telomeres such as EM cells [12]. The other statistically significant correlation was an inverse correlation between blood TL and the number of CD38 molecules on CM cells. CD38 is a marker of cell activation than in other studies [13] has also been negatively associated with shorter blood TL.

The main limitations of our study are the small sample size that decreases power to find associations and the multiplicity of comparisons performed that increases the risk of finding false significant results. Another limitation is the lack of measurement of TL within each cell subset which would allow to know if the blood TL is weighted significantly by a specific cell type. Also, we only performed assessments at baseline and W96, which does not allow to determine relation between dynamics of immune recovery and/or control of immune activation and TL changes.

In summary, we found that first-line ART increased blood TL and that this observation is probably a combined consequence of CD4 immune reconstitution and a concomitant decreased immune CD8 cell activation.

## Supporting information

S1 Appendix Correlation between change of the CD4 and CD8 population markers and change in telomere length between W00 and W96 adjusted by telomere length at week 0 and age at baseline, n = 31. (DOCX)

S2 Appendix NEAT 001/ANRS 143 Study Group. (PDF)
