# Supplementary material for: Correlation between blood telomere length and CD4+ CD8+ T-cell subsets changes 96 weeks after initiation of antiretroviral therapy in HIV-1–positive individuals
Source: PLoS One. 2020 Apr 8;15(4):e0230772. doi: 10.1371/journal.pone.0230772 (PMC7141657; doi:10.1371/journal.pone.0230772)
Supplement: S1 Appendix — (DOCX) [file pone.0230772.s001.docx]

Table 1. Correlation between change of the CD4 and CD8 population markers and change in telomere length between W00 and W96, n = 31.

| **T cell population** | **TL change for an increase of 100 units of the marker* [95% confidence interval]** | **p-value** |
| --- | --- | --- |
| **CD4^+^ (%)** |  |  |
| Total CD4^+^ | 0.46 [0.3 ; 0.89] | 0.04 |
| E cells | -0.27 [-0.55 ; 0.01] | 0.06 |
| EM cells  t | -0.55 [-1.00 ; -0.10] | 0.02 |
| TEMRA cells | 0.20 [-0.01 ; 0.41] | 0.06 |
| **CD8^+^ (%)** |  |  |
| Total CD8^+^ | -0.38 [-0.87 ; 0.10] | 0.11 |
| CD38^+^ | -0.21 [-0.45 ; 0.03] | 0.09 |
| HLA-DR^+^ CD38^+^ Naïve cells | -2.00 [-3.74 ; -0.25] | 0.03 |
| CD38^+^ PE molecules on CM cells | -0.001 [-0.002 ; 0.000] | 0.05 |
| CD38^+^ CCR5+ HLA-DR^+^ CM cells | -0.45 [0.98 ; 0.09] | 0.10 |
| CD38^+^ PE molecules on E cells | -0.002 [-0.004 ; 0.000] | 0.06 |
| CD38^+^ PE molecules on TM cells | -0.003 [-0.006 ; 0.000] | 0.10 |

* Adjusted by telomere length at week 0 and age at baseline
