# Supplementary material for: Correlation between blood telomere length and CD4+ CD8+ T-cell subsets changes 96 weeks after initiation of antiretroviral therapy in HIV-1–positive individuals
Source: PLoS One. 2020 Apr 8;15(4):e0230772. doi: 10.1371/journal.pone.0230772 (PMC7141657; doi:10.1371/journal.pone.0230772)
Supplement: S2 Appendix — (PDF) [file pone.0230772.s002.pdf]

Asterisk (*) indicates staff who left during the trial.

Trial Development Team (TDT):

- **Belgium**: Nikos Dedes (Brussels)
- **France**: Genevieve Chene, Laura Richert (Bordeaux), Clotilde Allavena, Francois Raffi (Nantes) and Brigitte Autran (Paris)
- **Italy**: Andrea Antinori, Raff aella Bucciardini and Stefano Vella (Rome)
- **Poland**: Andrzej Horban (Warsaw)
- **Spain**: Jose Arribas (Madrid)
- **UK**: Abdel G Babiker, Marta Boffito, Deenan Pillay and Anton Pozniak (London)

Trial Steering Committee (TSC):

- **Belgium**: Xavier Franquet* and Siegfried Schwarze (Brussels)
- **Denmark**: Jesper Grarup (Copenhagen)
- **France**: Genevieve Chene, Aurelie Fischer*, Laura Richert, Cedrick Wallet (Bordeaux), Francois Raffi (Nantes), Alpha Diallo, Jean-Michel Molina, and Juliette Saillard (Paris)
- **Germany**: Christiane Moecklinghoff (Janssen Pharmaceuticals; Neuss) and Hans-Jurgen Stellbrink (Hamburg)
- **Italy**: Stefano Vella (Rome)
- **Netherlands**: Remko Van Leeuwen (Amsterdam)
- **Spain**: Jose Gatell (Barcelona)
- **Sweden**: Eric Sandstrom (Stockholm)
- **Switzerland**: Markus Flepp (Zurich)
- **UK**: Abdel G Babiker, Fiona Ewings*, Elizabeth C George, Fleur Hudson, and Anton Pozniak (London)
- **USA**: Gillian Pearce*, Romina Quercia*, Felipe Rogatto (Gilead Sciences; Foster City, CA), Randi Leavitt, and Bach-Yen Nguyen* (Merck Laboratories; Whitehouse Station, NJ).

Independent Data Monitoring Committee (IDMC):

- Tim Peto (Chair), Oxford, UK
- Frank Goebel, Munich, Germany
- Simone Marcotullio, Rome, Italy
- Veronica Miller, Washington DC, USA.
- Peter Sasieni, London, UK

Trial Management Team (TMT):

- **France**: Clotilde Allavena and François Raffi (Nantes)
- **Italy**: Stefano Vella (Rome)
- **UK**: Anton Pozniak (London)

- **CMG-EC, INSERM U897 Coordinating Unit, Bordeaux, France:**

Geneviève Chêne, Head of coordinating CTU, Member, Bordeaux, France
Fabien Arnault*, Coordinating CTU representative, Member, Bordeaux, France
Céline Boucherie*, Bordeaux CTU representative, Observer, Bordeaux, France
Aurélie Fischer*, Coordinating CTU representative, Member, Bordeaux, France
Delphine Jean*, Bordeaux CTU representative, Observer, Bordeaux, France
Virginie Paniego*, Coordinating CTU representative, Member, Bordeaux, France
Felasoa Paraina, Bordeaux CTU representative, Observer, Bordeaux, France
Laura Richert, Coordinating CTU representative, Member, Bordeaux, France
Elodie Rouch*, Bordeaux CTU representative, Observer, Bordeaux, France
Christine Schwimmer, Coordinating CTU representative, Member, Bordeaux, France
Malika Soussi*, Bordeaux CTU representative, Observer, Bordeaux, France
Audrey Taieb*, Bordeaux CTU representative, Observer, Bordeaux, France
Monique Termote, Coordinating CTU representative, Member, Bordeaux, France
Guillaume Touzeau*, Coordinating CTU representative, Member, Bordeaux, France
Cédrick Wallet, Bordeaux CTU representative, Member, Bordeaux, France

- **MRC Clinical Trials Coordinating Unit, London, UK:**

Abdel G Babiker, Trial Statistician, Member, London, UK
Adam Cursley, MRC CTU representative, Observer, London, UK
Wendy Dodds*, MRC CTU representative, Member, London, UK
Fiona Ewings*, Trial Statistician, Member, London, UK
Elizabeth C George, Trial Statistician, Member, London, UK
Anne Hoppe*, MRC CTU representative, Observer, London, UK
Fleur Hudson, MRC CTU representative, Member, London, UK
Ischa Kummeling*, MRC CTU representative, Observer, London, UK
Filippo Pacciarini*, MRC CTU representative, Observer, London, UK
Nick Paton*, MRC CTU representative, Observer, London, UK
Charlotte Russell, MRC CTU representative, Observer, London, UK
Kay Taylor*, MRC CTU representative, Observer, London, UK
Denise Ward, MRC CTU representative, Observer, London, UK

- **CHIP Coordinating Unit, Copenhagen, Denmark:**

Bitten Aagaard*, CHIP CTU representative, Observer, Copenhagen, Denmark
Marius Eid, CHIP CTU representative, Observer, Copenhagen, Denmark
Daniela Gey*, CHIP CTU representative, Member, Copenhagen, Denmark
Birgitte Gram Jensen*, CHIP CTU representative, Observer, Copenhagen, Denmark
Jesper Grarup, CHIP CTU representative, Member, Copenhagen, Denmark
Marie-Louise Jakobsen*, CHIP CTU representative, Observer, Copenhagen, Denmark
Per O. Jansson, CHIP CTU representative, Member, Copenhagen, Denmark
Karoline Jensen*, CHIP CTU representative, Member, Copenhagen, Denmark
Zillah Maria Joensen, CHIP CTU representative, Observer, Copenhagen, Denmark

Ellen Moseholm Larsen*, CHIP CTU representative, Observer, Copenhagen, Denmark
Christiane Pahl*, CHIP CTU representative, Observer, Copenhagen, Denmark
Mary Pearson*, CHIP CTU representative, Member, Copenhagen, Denmark
Birgit Riis Nielsen, CHIP CTU representative, Observer, Copenhagen, Denmark
Søren Stentoft Reilev*, CHIP CTU representative, Observer, Copenhagen, Denmark

- **Amsterdam Medical Center Coordinating Unit, Amsterdam, The Netherlands:**

Ilse Christ, AMC CTU representative, Observer, Amsterdam, The Netherlands
Desiree Lathouwers*, AMC CTU representative, Member, Amsterdam, The Netherlands
Corry Manting, AMC CTU representative, Member, Amsterdam, The Netherlands
Remko Van Leeuwen, AMC CTU representative, Member, Amsterdam, The Netherlands

- **ANRS, Paris, France:**

Alpha Diallo, Pharmacovigilance representative, Member, Paris, France
Bienvenu Yves Mendy*, Pharmacovigilance representative, Member, Paris, France
Annie Metro*, Pharmacovigilance representative, Member, Paris, France
Juliette Saillard, Sponsor representative, Member, Paris, France
Sandrine Couffin-Cadiergues, Sponsor representative, Observer, Paris, France

- **ISS, Rome, Italy:**

Anne-Laure Knellwolf*, NEAT management representative, Observer, Rome, ltaly
Lucia Palmisiano, NEAT management representative, Member, Rome, ltaly

Local CTUs:

- **GESIDA, Madrid, Spain:**

Esther Aznar, Cristina Barea*, Manuel Cotarelo*, Herminia Esteban, Iciar Girbau*, Beatriz Moyano, Miriam Ramirez*, Carmen Saiz, Isabel Sanchez, Maria Yllescas

- **ISS, Rome, Italy:**

Andrea Binelli, Valentina Colasanti, Maurizio Massella, Lucia Palmisiano.

- **University of Athens Medical School, Greece:**

Olga Anagnostou, Vicky Gioukari, Giota Touloumi.

Study Investigators:

- **Austria:** Brigitte Schmied (National Coordinating Investigator), Armin Rieger, Norbert Vetter

- **Belgium:** Stephane De Wit (National Coordinating Investigator), Eric Florence, Linos Vandekerckhove

- **Denmark:** Jan Gerstoft (National Coordinating Investigator), Lars Mathiesen

- **France:** Christine Katlama (National Coordinating Investigator), Andre Cabie, Antoine Cheret, Michel Dupon, Jade Ghosn*, Pierre-Marie Girard, Cécile Goujard, Yves Lévy, Jean-Michel Molina, Philippe Morlat, Didier Neau, Martine Obadia, Philippe Perre, Lionel Piroth, Jacques Reynes, Pierre Tattevin, Francois Raffi, Jean Marie Ragnaud*, Laurence Weiss, Yazdanpanah Yazdan*, Patrick Yeni, David Zucman

- **Germany:** Georg Behrens (National Coordinating Investigator), Stefan Esser, Gerd Fätkenheuer, Christian Hoffmann, Heiko Jessen, Jürgen Rockstroh, Reinhold Schmidt, Christoph Stephan, Stefan Unger

- **Greece:** Angelos Hatzakis (National Coordinating Investigator), George L Daikos, Antonios Papadopoulos, Athamasios Skoutelis

- **Hungary:** Denes Banhegyi (National Coordinating Investigator)

- **Ireland:** Paddy Mallon (National Coordinating Investigator), Fiona Mulcahy

- **Italy:** Andrea Antinori (National Coordinating Investigator), Massimo Andreoni, Stefano Bonora, Francesco Castelli, Antonella D'Arminio Monforte, Giovanni Di Perri, Massimo Galli, Adriano Lazzarin, Francesco Mazzotta, Carlo Torti *, Vincenzo Vullo

- **The Netherlands:** Jan Prins (National Coordinating Investigator), Clemens Richter, Dominique Verhagen, Arne Van Eeden*

- **Poland:** Andrzej Horban (National Coordinating Investigator)

- **Portugal:** Manuela Doroana (National Coordinating Investigator), Francisco Antunes*, Fernando Maltez, Rui Sarmento-Castro,

- **Spain:** Juan Gonzalez Garcia (National Coordinating Investigator), José López Aldeguer, Bonaventura Clotet, Pere Domingo, Jose M Gatell, Hernando Knobel, Manuel Marquez, Martin Pilar Miralles, Joaquin Portilla, Vicente Soriano, Maria-Jesus Tellez

- **Sweden:** Anders Thalme (National Coordinating Investigator), Anders Blaxhult, Magnus Gisslen

- **UK:** Alan Winston (National Coordinating Investigator), Julie Fox, Mark Gompels, Elbushra Herieka, Margaret Johnson, Clifford Leen, Anton Pozniak, Alastair Teague, Ian Williams

Endpoint Review Committee (ERC):

- **Australia**: Mark Alastair Boyd, (Sydney)

- **Denmark**: Jesper Grarup, Per O Jansson, Nina Friis Møller, and Ellen Frøsig Moseholm Larsen (Copenhagen)
- **France**: Philippe Morlat (Bordeaux), Lionel Piroth (Dijon), and Vincent Le Moing (Montpellier)
- **Netherlands**: Ferdinand W N M Wit, chair (Amsterdam)
- **Poland**: Justyna Kowalska (Warsaw)
- **Spain**: Juan Berenguer and Santiago Moreno (Madrid)
- **Switzerland**: Nicolas J Müller (Zurich)
- **UK**: Estée Török (Cambridge), Frank Post (London), and Brian Angus (Oxford)

Sub-study working groups:

- **Virology working group:**

Vincent Calvez (coordinator), Charles Boucher, Simon Collins, David Dunn (statistician), Sidonie Lambert, Anne-Geneviève Marcelin, Carlo Federico Perno, Deenan Pillay, Ellen White (statistician)

- **Pharmacology and adherence working group:**

Marta Boffito (coordinator), Adriana Ammassari, Andrea Antinori, Wolgang Stoehr (statistician)

- **Immunology working group:**

Brigitte Autran (coordinator), Reinhold Ernst Schmidt, Michal Odermarsky, Colette Smith, Rodolphe Thiébaut (statistician)

- **Toxicity, including co-infection working group:**

Jose Arribas (coordinator), Jose Ignacio Bernardino De La Serna, Antonella Castagna, Stephane De Wit, Xavier Franquet, Hans-Jackob Furrer, Christine Katlama, Amanda Mocroft (statistician), Peter Reiss

- **Quality of life working group:**

Raffaella Bucciardini (coordinator), Nikos Dedes, Vincenzo Fragola, Elizabeth C George (statistician), Marco Lauriola, Rita Murri, Pythia Nieuwkerk, Bruno Spire, Alain Volny-Anne, Brian West

- **Neurocognitive function working group:**

Hélène Amieva (coordinator), Andrea Antinori, Josep Maria Llibre Codina, Laura Richert, Wolgang Stoehr (statistician), Alan Winston

- **Pharmaco-economics working group:**

Francesco Castelli (coordinator), Marco Braggion (statistician), Emanuele Focà
